# Sociodemographic, Clinical, and Behavioral Factors Associated with Sexual Transmitted Infection among HIV-1 Positive Migrants in Portugal: Are There Differences between Sexes?

**DOI:** 10.3390/pathogens13070598

**Published:** 2024-07-19

**Authors:** Mafalda N. S. Miranda, Victor Pimentel, Jacqueline Graça, Sofia G. Seabra, Cruz S. Sebastião, António Diniz, Domitília Faria, Eugénio Teófilo, Fausto Roxo, Fernando Maltez, Isabel Germano, Joaquim Oliveira, José Ferreira, José Poças, Kamal Mansinho, Luís Mendão, Maria João Gonçalves, Margarida Mouro, Nuno Marques, Patrícia Pacheco, Paula Proença, Raquel Tavares, Ricardo Correia de Abreu, Rosário Serrão, Telo Faria, BESTHOPE Study Group, M. Rosário O. Martins, Perpétua Gomes, Ana B. Abecasis, Marta Pingarilho

**Affiliations:** 1 Global Health and Tropical Medicine, GHTM, Associate Laboratory in Translation and Innovation towards Global Health, LA-REAL, Instituto de Higiene e Medicina Tropical, IHMT, Universidade NOVA de Lisboa, UNL, Rua da Junqueira 100, 1349-008 Lisbon, Portugal; mafaldansmiranda@ihmt.unl.pt (M.N.S.M.); cruzsebastiao@ihmt.unl.pt (C.S.S.);; 2 Unidade Imunodeficiência, Hospital Pulido Valente—Unidade Local de Saúde Santa Maria, 1769-001 Lisbon, Portugal; 3 Serviço de Medicina 3, Hospital de Portimão—Unidade Local de Saúde Algarve, 8500-338 Portimão, Portugal; 4 Serviço de Medicina 2.3, Hospital de Santo António dos Capuchos, Centro Hospitalar de Lisboa Central—Unidade Local de Saúde de São José, 1169-050 Lisbon, Portugal; 5 Unidade de Doenças Infecciosas, Hospital de Santarém—Unidade Local de Saúde Lezíria, 2005-177 Santarém, Portugal; 6 Serviço de Doenças Infeciosas, Hospital Curry Cabral, Centro Hospitalar Universitário Lisboa Central—Unidade Local de Saúde São José, 1069-166 Lisbon, Portugal; 7 Instituto de Saúde Ambiental, Faculdade de Medicina, Universidade de Lisboa, 1649-026 Lisbon, Portugal; 8 Serviço de Medicina 1.4, Hospital de São José, Centro Hospitalar Universitário de Lisboa Central—Unidade Local de Saúde São José, 1150-199 Lisbon, Portugal; 9 Serviço de Prevenção e Controlo de Infeções e de Resistências aos Antimicrobianos—Unidade Local de Saúde de Coimbra, 3004-561 Coimbra, Portugal; 10 Serviço de Medicina 2, Hospital de Faro—Unidade Local de Saúde Algarve, 8000-386 Faro, Portugal; 11 Serviço de Infeciologia, Centro Hospitalar de Setúbal—Unidade Local de Saúde Arrábida, 22910-446 Setúbal, Portugal; 12 Serviço de Doenças Infeciosas, Hospital de Egas Moniz, Centro Hospitalar de Lisboa Ocidental, 1349-019 Lisbon, Portugal; 13 Grupo de Ativistas em Tratamentos (GAT), 1000-228 Lisbon, Portugal; 14 Serviço de Infeciologia, Centro Hospitalar do Porto, 4099-001 Porto, Portugal; 15 Serviço de Infeciologia, Hospital de Aveiro, Centro Hospitalar Baixo Vouga, 3810-164 Aveiro, Portugal; 16 Serviço de Infeciologia, Hospital Garcia da Orta, 2805-267 Almada, Portugal; 17 Serviço de Infeciologia, Hospital Dr. Fernando da Fonseca, 2720-276 Amadora, Portugal; 18 Serviço de Infeciologia, Hospital de Faro—Unidade Local de Saúde Algarve, 8000-386 Faro, Portugal; 19 Serviço de Infeciologia, Hospital Beatriz Ângelo, 2674-514 Loures, Portugal; 20 Serviço de Doenças Infeciosas, Hospital Pedro Hispano—Unidade de Local de Saúde de Matosinhos, 4464-513 Matosinhos, Portugal; 21 Serviço de Doenças Infeciosas, Unidade Local de Saúde de São João, 4202-451 Porto, Portugal; 22 Hospital José Joaquim Fernandes—Unidade Local de Saúde do Baixo Alentejo, 7801-849 Beja, Portugal; 23 Laboratório de Biologia Molecular (LMCBM, SPC, ULSLO-HEM), 1349-019 Lisbon, Portugal; 24 Egas Moniz Center for Interdisciplinary Research (CiiEM), Egas Moniz School of Health & Science, 2829-511 Caparica, Portugal

**Keywords:** HIV-1, newly infected patients, migrants, sexual risk behavior, STIs

## Abstract

Introduction: Sexually transmitted infections (STIs) continue to occur at high levels. According to the WHO, each year there are an estimated 374 million new infections with syphilis, gonorrhea, chlamydia, and trichomoniasis. STIs are associated with an increased risk of acquiring HIV infection. Migrants are reportedly highly affected by STIs. Objectives: This study aims to characterize factors associated with STIs in a population of HIV-positive migrants living in Portugal. Methodology: This is a cross-sectional observational study of 265 newly diagnosed HIV-1 positive migrants, who were defined as individuals born outside Portugal. This group of people were part of the BESTHOPE study that was developed in 17 Portuguese hospitals between September 2014 and December 2019, and included information collected through sociodemographic and behavioral questionnaires filled in by the migrant patients, clinical questionnaires filled in by the clinicians and HIV-1 genomic sequences generated through resistance testing (Sanger sequencing). A multivariable statistical analysis was used to analyze the association between sociodemographic characteristics, sexual behaviors, HIV testing and sexual infections. Results: Most HIV-1 positive individuals included in the study were men (66.8%) and aged between 25 and 44 years old (59.9%). Men had a higher proportion of STIs when compared to women (40.4% vs. 14.0%) and the majority of men reported homosexual contacts (52.0%). Most men reported having had two or more occasional sexual partners in the previous year (88.8%) and 50.9% reported always using condoms with occasional partners, while 13.2% never used it. For regular partners, only 29.5% of the women reported using condoms, compared to 47.3% of men. Other risk behaviors for acquiring HIV, such as tattooing and performing invasive medical procedures, were more prevalent in men (38.0% and 46.2%, respectively), when compared to women (30.4% and 45.1% respectively) and 4.7% of men reported having already shared injectable materials, with no data for comparison in the case for women. Additionally, 23.9% of women reported having had a blood transfusion while only 10.3% of men reported having had this medical procedure. Meanwhile, 30.9% of the individuals reported having been diagnosed with some type of STI in the last 12 months. In addition, 43.3% of individuals that answered a question about hepatitis reported to be infected with hepatitis B, while 13.0% reported having hepatitis C infection. According to the multivariable analysis, the only transmission route was significantly associated with reports of previous STI infection: men who have sex with men (MSM) were 70% more likely to have been diagnosed with an STI in the past 12 months compared to the heterosexual route. Conclusion: HIV-1 infected men were more likely to report previous STIs than women. On the other hand, most migrant women had a regular sexual partner and never or only sometimes used condoms. This somewhat discrepant findings suggest that gender inequalities may make women unable to negotiate safe sexual practices, resulting in increased susceptibility to infection. However, since migrant women report less STIs, we cannot exclude that these STIs may remain undiagnosed. The implementation of safer sex awareness campaigns for condom use and screening for STIs in women is crucial. On the other hand, health education campaigns for STI knowledge need to be implemented for both MSM and women and their partners.

## 1. Introduction

Human immunodeficiency virus (HIV) infection is still one of the biggest global public health problems with around 39 million people living with HIV (PLHIV) worldwide. In 2022, around 1.3 million new infections were diagnosed; 70% of these occurred among vulnerable groups [1]. To exacerbate this issue, coinfection with sexually transmitted infections (STI) is a serious concern. Approximately 374 million new cases of STIs (syphilis, gonorrhea, chlamydia and trichomoniasis) occur annually, especially in people aged 15–49 years [2]. 

Migrant populations including those from African countries are disproportionately affected by infectious diseases such as HIV and other STIs [3]. Migrants are considered a vulnerable population, since in general, they experience vulnerability with restricted access to health care, poverty, low levels of education, cultural and language barriers, and stigma. These factors make migrants more susceptible to acquiring infectious diseases including HIV [4].

Moreover, there were identified multiple risk factors in the literature associated with HIV/STI co-infection, such as having a high number of different sexual partners, sexual contact with casual partners and sex workers, inconsistent condom use, and the ingestion of drugs and alcohol before sexual contact [5]. The treatment and prevention of these STIs interrupt the chain of transmission and prevent possible complications [6]. Health education and adherence to safe sex practices like the use of condoms and reducing the number of sexual partners are essential elements in preventing STIs [7]. Additionally, having an STI increases the infectiousness of people living with HIV. Furthermore, this increase in infectiousness leads to a potential for HIV acquisition in people at risk for HIV [8]. 

In Portugal, the rate of migration continues to rise, including many immigrants from Portuguese-speaking countries. In 2021, the majority arrived from Brazil, and a large part arrived from Portuguese-speaking African countries [9]. Moreover, in 2021, almost half (47%) of HIV new diagnoses in Portugal were among foreign-born individuals; 21.5% of those occur in individuals with origin in sub-Saharan African countries [10]. 

In Portugal, specifically in Porto and Lisbon Metropolitan Areas, the most commonly reported STIs were chlamydia, gonorrhea and syphilis, the latter having the highest rate of notifications [11]. For chlamydia, the highest incidence of cases was among 25 to 34 years old, for gonorrhea the highest incidence was among adolescent males and young adults aged between 15 and 24 years old, while for syphilis it was found mostly in males between 45 and 64 years old [11,12].

Given the high increase in migrants living in Portugal in recent years [13] and the fact that STIs are usually associated with an increased risk of acquiring HIV infection, it is important to understand the main factors associated with STI infection in HIV-1 positive individuals in migrant populations living in Portugal.

## 2. Materials and Methods

### 2.1. Study Population and Data Collection

The protocol was in accordance with the declaration of Helsinki and approved by the Ethical Committee of all hospitals involved in the study (see Supplementary Table S1 in the Supplementary Materials).

Data from 265 newly diagnosed HIV-1 positive migrants (defined as a person who was born outside Portugal) were collected in 17 hospitals located in Portugal between September 2014 and December 2019. These migrants are part of the BESTHOPE database containing anonymized individuals’ information, including demographic, clinical, behavioral and genotype resistance data. All People Living with HIV-1 (PLWH) data were generated in the context of routine clinical care. Informed consent was obtained from all subjects involved in the study.

The BESTHOPE study was an observational cross-sectional study that collected data of people living with HIV-1 followed in Portuguese hospitals through different data collection instruments, such as socio-behavioral questionnaires, clinical questionnaires and genomic sequences of the HIV-1 virus, to understand the dynamics and the behavioral determinants of HIV transmission.

The data were collected with a survey questionnaire in the infectious diseases/internal medicine consultations constructed by researchers in collaboration with patients and NGO members, and then completed by the participants. Clinicians provided clinical data (https://methods.sagepub.com/case/clinical-sociobehavioral-questionnaires-genomic-data-hiv-transmission, accessed on 8 July 2024). 

### 2.2. Management and Statistical Analysis of Data

Data were entered, cleaned, coded and checked for missing values, outliers and inconsistencies. Descriptive analysis was used to characterize the sociodemographic, clinical and behavioral characteristics of the individuals included in the study. The association between sex (the outcome) and the qualitative variables was analyzed using Chi-square (X2) or Fisher tests. To compare the continuous variables, such as age, between the two groups, the Mann–Whitney U test was used. To analyze the associations between sociodemographic characteristics, sexual behaviors, HIV testing and sexual infections a multivariable analysis was used using logistic regression to obtain an adjusted odds ratio (aOR) and respective 95% confidence interval (CI).

Factors which were associated with outcomes at the 20% significance level in the bivariate models were included in the multivariable analysis. All analyses were conducted in SPSS Statistic software version 27. For the multivariable analysis, the variable “country of origin” was classified into (a) sub-Saharan Africa, (b) Brazil and Latin America, (c) Western Europe and Eastern Europe, and (d) other countries. The variable “employment situation” was classified as (1) employed and (2) not employed, the latter including the unemployed, retired or students.

## 3. Results

### 3.1. Epidemiologic and Clinical Data

The sociodemographic characteristics of the study participants are summarized in Table 1. Most HIV-1 positive individuals included in the study were males (66.8%) and aged between 25 and 44 years old (59.9%). Regarding marital status and level of education, in our population more than half of individuals were single (56.3%) and about a third (30.9%) finalized secondary school level. Most individuals were employed (72.7%) and half (50.0%) reported their financial situation as being “sufficient”. Single individuals were mostly male, while married individuals were mostly women (*p* = 0.017). Some differences were found between women’s and men’s perceptions, since regarding their current income, 46.8% of women reported their financial situation as being insufficient, while 60.6% of men considered it as sufficient (*p* < 0.001). Regarding the country of origin, 49.8% were originated from sub-Saharan African countries (including Angola (31.1%), Cape Verde (20.5%) and Guinea-Bissau (37.1%)), and 39.6% of individuals had Brazilian origin. The men were mostly Brazilian (48.6%) while the women were of African origin (72.7%), and within the latter, the majority were Guineans (46.9%) (*p* < 0.001).

### 3.2. Behavioral Data and Risk Factor

Table 2 presents data on sexual risk behaviors and other risk factors associated with HIV infection.

Regarding the type of sexual relationships, data showed that men have a high proportion of homosexual contacts (52.8%) when compared to heterosexual contacts (38.9%). Bisexuals, individuals who had both men and women as sexual partners, represented only 5.3% of the total number of study participants. Regarding the type of partner in the last sexual intercourse, most men (54.5%) described having had a regular partner. 

A high proportion of women and men (87.9% and 75.0%, respectively) had a single regular sexual partner in the last 12 months, with no significant differences between sex (*p* = 0.223).

On the other hand, when we compared the proportion of occasional sexual partners in the last year, we found that most men had two or more occasional sexual partners (88.8%). For women, there was only one response for this variable, so it was not possible to establish a comparison between both sexes. Moreover, for men having occasional sexual partners, 50.9% always used condoms with those partners. Only three women reported having always used a condom with occasional sexual partners. Other risk behaviors for acquiring HIV, such as tattooing and performing invasive medical procedures, were more prevalent in men, but not statistically significant (38.0% and 46.2%, respectively), when compared to women (30.4% and 45.1% respectively) and 4.7% of men reported having already shared injectable materials, while all of the 45 women who responded to this question reported not having shared needles. Additionally, 23.9% of women reported having had a blood transfusion while only 10.3% of men reported having had this medical procedure (*p* = 0.044).

### 3.3. Testing for HIV and Sexually Transmitted Infections 

Information regarding HIV testing and sexually transmitted infections is shown in Table 3.

The evaluation of data on HIV infection shows that the main route of transmission of the infection was sexual (77.2%), with a higher proportion of men reporting sexual transmission compared to women (82.2% and 68.4%, respectively). A large proportion of persons did not know what the transmission route had been (19.6%). Moreover, most women reported being infected by regular partners and most men reported being infected by occasional partners (82.4% and 63.6%, respectively, *p* < 0.001).

Overall, 30.9% of the individuals were diagnosed with some type of STI other than HIV in the last 12 months. Hepatitis B was diagnosed in 43.3% of individuals, while hepatitis C was diagnosed in 13.0% of individuals. 

The data also revealed that most study individuals were under an antiretroviral therapy regimen (74.5%), although most were not aware if their sexual partners were also under antiretroviral therapy (72.2%).

### 3.4. Determinants Associated with Sexually Transmitted Infections 

The factors associated with patients who had an STI diagnosis in the last 12 months are shown in Table 4.

Unadjusted ORs suggested that female individuals and married individuals compared to single ones were less likely to have an STI. On the other hand, individuals reporting heterosexual route of transmission compared to those with MSM route of transmission, individuals with origins in Brazil and other Latin American countries when compared to individuals with origins in sub-Saharan Africa and individuals with secondary or higher education compared to third level education were more likely to have an STI. 

A univariate analysis was also conducted including behavioral variables in the model, but when computing the multivariable analysis, the model was not possible to execute. However, we observed that the variables related to acquiring an STI were the “sexual partner” where women were less likely to being infected with an STI then men, having an occasional sexual partner in the last sexual relation compared to regular sexual partner, having between two and three regular sexual partners in the last 12 months compared to having one, never using condom with sex workers compared to always using and having tattoos compared to not having them.

In the multivariable analysis, sex, country of origin, civil status, transmission route and school level were included. However, results showed that only transmission route was associated with an STI diagnosis in the past 12 months. The MSM route of transmission was more likely to be associated with an STI diagnosis (in the past 12 months) when compared to heterosexual route (ORa: 5.695; 95% CI: 1.249–25.978; *p* = 0.025).

## 4. Discussion

This study provides valuable data about the epidemiological profile and risk factors associated with STIs in the migrant population residing in Portugal, emphasizing the differences between female and male migrant populations. One main finding of this study was the suggested gender inequalities that may make migrant women more vulnerable and unable to negotiate safe sexual intercourse practices and therefore being more susceptible to acquiring STIs. These findings resulted from the behavioral questionnaire where migrant women reported having a regular sexual partner and never or only sometimes used condoms; on the other hand, HIV-1 infected men were more likely to report previous. Although, as you can see below, from the study population, males were the bigger sample comparing sexes.

Among the 265 study participants, 66.8% were male and 59.9% were in the 25–44 age group. More than half of the participants were single (56.3%) and most were men (65.7%; *p* = 0.017). In another study carried out in Portugal in 2015 to examine the risk behaviors and determinants associated with HIV infection among the migrant population, more than half of the participants (58.0%) were males, with a median age of 37 years and 52.3% were single; those results were in accordance with our study [14]. 

In this study, we compared the current occupation and financial situation between both sexes since it is known to be a very discussed topic in the general population and even more among migrants. Although, we observed that a large proportion of the individuals were employed (72.7%), but no significant differences in professional status were observed between males and females. Regarding their financial situation, half of the participants (50.0%) evaluated their financial situation as sufficient, of whom 60.6% were males. In the majority (46.8%), women evaluated their professional situation as insufficient, which was expected. The same was observed in another Portuguese study where 80.1% of the participants were employed and 50.2% assessed their economic situation as sufficient or more than sufficient; however, 55.5% of the women assessed their economic situation as very insufficient or insufficient [15]. 

Assessing the country of origin in our study is of most importance because the ratio of migrants from different origins differs between sexes. We could observe in our study that most females were born in sub-Saharan African countries (72.7%); however, males were mostly from Brazil (48.6%). These findings are in accordance with results from another study that aimed to determine the prevalence of HIV testing and its associated factors in a sample of migrants in Portugal, which showed that 53.4% of the participants were males, 50.5% of the participants were from Latin America, being 99.3% of them from Brazil, and 34.8% were African, mostly from sub-Saharan Africa [15].

In our study, the clinical questionnaire reported a large proportion of individuals from our study as being heterosexual (59.3%) and on the behavioral and self-fulfilled questionnaire, all migrant women reported a male sexual partner and 38.9% of men reported a female sexual partner. Furthermore, the sexual partner in the sexual relation was reported to be mostly regular in both men and women (54.5% and 90.9%, respectively). However, men had a higher proportion of occasional sexual partners compared to women (43.2% and 9.1%, respectively). The number of occasional sexual partners in the past 12 months was 88.8% for men who had more than two partners. Such results were in accordance with other studies [16,17], which indicated a high rate of unsafe sexual behavior among migrants. Besides having more than one occasional sexual partner, those behaviors also included irregular condom use. For women, of the four reporting having had an occasional sexual partner in the last sexual intercourse, only one answered the question about the number of occasional sexual partners (one sexual occasional partner).

More findings from our study showed that migrant women who reported mostly having only one regular sexual partner use condoms less often compared to men who frequently reported having several occasional sexual partners. Yet, the association between condom use and sex was not statistically significant. Other studies have shown the same results, where women have, in general, a lower frequency of condom use with regular partners [16,17]. This could be motivated on one hand by the desire to get pregnant, on the other side by a greater difficulty in negotiating safe sex with their partners, low levels of knowledge about HIV, lack of dialogue about HIV with partners and low levels of education [18,19]. A study of 254 women at high risk of acquiring HIV infection in KwaZulu-Natal, South Africa showed that although they had sufficient knowledge about HIV transmission and prevention, there was no association with condom use with regular partners. The difficulty in negotiating condom use with regular partners is thought to be associated with perception of lack of trust or infidelity by the partner. On the other hand, using condoms was more acceptable if it was the only contraception used, rather than a means of preventing transmission of an infection [20]. In a Portuguese study involving 790 migrants from sub-Saharan Africa, inconsistent condom use was more prevalent among women, despite the majority having only one sexual partner, compared to men. This factor was shown to be independent of age, level of education, occupation, length of stay in Portugal and experience of violence or forced sex [21]. Other factors that may increase women’s vulnerability to HIV acquisition include behavioral, socioeconomic and cultural factors, one example being the patriarchal culture of society, especially in developing countries such as countries in sub-Saharan Africa. In this region, women’s vulnerability is exacerbated which deprives women of the power of self-protection against HIV infection [22]. Although there was a lower proportion of condom use among women, men may have higher rates of risk behaviors, including a higher percentage of sexual intercourse with occasional partners and having more than one casual partner in the last year. These factors may also underlie a higher proportion of STIs among males, as there is evidence that increased STIs are associated with increased HIV transmission [16].

After analyzing the differences between sexes related to their behaviors and sexual activities, we conducted an adjusted logistic regression model with the objective of understanding the risk factors that could be associated with acquiring STIs in this population. In our model, the only variable associated with migrants having been diagnosed with an STI was the transmission route, with men who reported MSM contact being more likely to have been diagnosed with an STI in the past 12 months compared to heterosexual contact. These results are in accordance with other studies that indicate sexual contact, mostly MSM contact, as one of the factors associated with STI diagnosis, especially in younger individuals [23,24]. In addition to transmission route, other factors associated with a higher probability of STI diagnosis have been described in the literature, such as single individuals, males, having a higher level of education, having more than one sexual partner and inconsistent condom use [23,25].

Because of the short- and long-term consequences of STIs and their close association with HIV acquisition, it is necessary to know the risk factors related to the increased likelihood of acquiring an STI. Factors such as earlier sexual debut and having multiple sexual partners are factors associated with an increased risk of acquiring an STI [26]. Other factors that may also influence STI acquisition include host susceptibility, the virulence of the pathogen, its concentration in seminal or genital fluid, the concomitant presence of other STIs, the vaginal ecosystem, male circumcision, condom, microbicide and contraceptive use, and risky sexual practices such as unprotected anal sex [26].

### Limitations

Our study analyzed clinical and behavioral data from HIV-1 infected migrants that were followed up in Portuguese hospitals. Since the core information of the study was collected through a self-fulfilled questionnaire, we could have a bias in the information fulfilled and that presents the major limitation of this study. This self-reported questionnaire poses a limitation in various ways; the first is the responses might be biased due to stigma still existing when the topic is HIV; the second is the language barrier; the questionnaire was in Portuguese and some migrants had some difficulties in understanding some questions and therefore it was the clinician that helped to fulfill it. We could also add to the limitations the memory bias, since some questions were related to passed situations and therefore we could have had answers that were not entirely correct. 

Another limitation of the study was the reduced sample size of the study population, which could not be representative of the migrants diagnosed with HIV-1 in Portugal. Our sampling corresponds to a sampling rate of around 17% of the total of new diagnosis in migrants in Portugal within these 5 years. We expected to achieve a higher sampling rate; however, the availability of hospitals to include patients was not always ideal.

## 5. Conclusions

Overall, this study concludes that migrants are exposed to several risk factors that increase their susceptibility to HIV infection and STI acquisition. Among these, migrant women are particularly exposed. In this case, the implementation of safer sex awareness campaigns for condom use and awareness of STI knowledge would need to be targeted to both migrant women and their partners.

## Figures and Tables

**Table 1:** Sociodemographic data of the participants included in the BESTHOPE study collected between September 2014 and December 2019.

Patient Characteristics	Total	Men	Women	*p* Value ^a^
	N (%)	N (%)	N (%)	
**Sex**	265 (100%)	177 (66.8%)	88 (33.2%)	
**Age group**	262 (100%)	175 (100%)	87 (100%)	
≤24	37 (14.1%)	26 (14.9%)	11 (12.6%)	*0.330*
25–44	157 (59.9%)	102 (58.3%)	55 (63.2%)	
45–63	62 (23.7%)	41 (23.4%)	21 (24.1%)	
≥ 64	6 (2.3%)	6 (3.4%)	---	
**Transmission Route**	263 (100%)	176 (66.9%)	87 (33.1%)	
Heterosexual	156 (59.3%)	72 (40.9%)	84 (96.6%)	***<0.001***
MSM	102 (38.8%)	102 (58.0%)	--	
Injectable Drug Users (IDU)	2 (0.8%)	2 (1.1%)	--	
Other/Unknown	3 (1.1%)	--	3 (3.4%)	
**Civil Status**	176 (100%)	108 (100%)	68 (100%)	
Divorced	19 (10.8%)	9 (8.3%)	10 (14.7%)	***0.017***
Married	56 (31.8%)	27 (25.0%)	29 (42.6%)	
Single	99 (56.3%)	71 (65.7%)	28 (41.2%)	
Widower	2 (1.1%)	1 (0.9%)	1 (1.5%)	
**School level**	175 (100%)	109 (100%)	66 (100%)	
None	6 (3.4%)	3 (2.8%)	3 (4.5%)	***0.134***
Third level (9th degree)	47 (26.9%)	23 (21.1%)	24 (36.4%)	
Secondary (12nd degree)	54 (30.9%)	35 (32.1%)	19 (28.8%)	
Advanced Technical	18 (10.3%)	11 (10.1%)	7 (10.6%)	
Higher education	50 (28.6%)	37 (33.9%)	13 (19.7%)	
**Current occupation**	172 (100%)	106 (100%)	66 (100%)	
Unemployed	29 (16.9%)	11 (10.4%)	18 (27.3%)	***0.046***
Employed	125 (72.7%)	82 (77.4%)	43 (65.2%)	
Retired	5 (2.9%)	4 (3.8%)	1 (1.5%)	
Student	11 (6.4%)	7 (6.6%)	4 (6.1%)	
Sex worker	2 (1.2%)	2 (1.9%)	---	
**Current income**	166 (100%)	104 (100%)	62 (100%)	
Very Insufficient	22 (13.3%)	10 (9.6%)	12 (19.4%)	***<0.001***
Insufficient	53 (31.9%)	24 (23.1%)	29 (46.8%)	
Sufficient	83 (50.0%)	63 (60.6%)	20 (32.3%)	
More than sufficient	8 (4.8%)	7 (6.7%)	1 (1.6%)	
**Country of origin**	265 (100%)	177 (100%)	88 (100%)	
Brazil	105 (39.6%)	86 (48.6%)	19 (21.6%)	***<0.001***
African countries	132 (49.8%)	68 (38.4%)	64 (72.7%)	
Angola	41 (31.1%)	22 (32.4%)	19 (29.7%)	
Cape Verde	27 (20.5%)	18 (26.5%)	9 (14.1%)	
Guinea Bissau	49 (37.1%)	19 (27.9%)	30 (46.9%)	
Other African Countries	15 (11.4%)	9 (13.2%)	6 (9.4%)	
Other countries *	28 (10.6%)	23 (12.9%)	5 (5.7%)	

* Eastern Europe, Western Europe and other Latin American countries; *p*-value ^a^: chi-square test or Fisher’s exact test.

**Table 2:** Behavioral data and risk factors of BESTHOPE study participants collected between September 2014 and December 2019.

	Total	Men	Women	*p* Value ^a^
	N (%)	N (%)	N (%)	
**Sexual partner**	172 (100%)	108 (100%)	61 (100%)	
Men	118 (69.8%)	57 (52.8%)	61 (100%)	***<0.001***
Women	42 (24.9%)	42 (38.9%)	---	
Men and Women	9 (5.3%)	9 (8.3%)	---	
**Sexual partner at last sexual relation**	132 (100%)	88 (100%)	44 (100%)	
Occasional	42 (31.8%)	38 (43.2%)	4 (9.1%)	***<0.001***
Regular	88 (66.7%)	48 (54.5%)	40 (90.9%)	
Sex worker	2 (1.5%)	2 (2.3%)	---	
**Number of regular sexual partners in last 12 months**	81 (100%)	48 (100%)	33 (100%)	
1 sexual partner	65 (80.2%)	36 (75.0%)	29 (87.9%)	*0.223*
2–3 sexual partners	13 (16.1%)	9 (18.8%)	4 (12.1%)	
≥4 sexual partners	3 (3.7%)	3 (6.2%)	---	
**Number of occasional sexual partners in last 12 months**	28 (100%)	27 (100%)	1 (100%)	
1 sexual partner	4 (14.3%)	3 (11.1%)	1 (100%)	***0.045***
2–3 sexual partners	12 (42.9%)	12 (44.4%)	---	
≥4 sexual partners	12 (42.9%)	12 (44.4%)	---	
**Condom use with regular sexual partner in the last 12 months**	118 (100%)	74 (100%)	44 (100%)	
Always	48 (40.7%)	35 (47.3%)	13 (29.5%)	***0.144***
Never	36 (30.5%)	19 (25.7%)	17 (38.6%)	
Sometimes	34 (28.8%)	20 (27.0%)	14 (31.8%)	
**Condom use with occasional sexual partner in the last 12 months**	61 (100%)	53 (100%)	8 (100%)	
Always	30 (49.2%)	27 (50.9%)	3 (37.5%)	*0.224*
Never	10 (16.4%)	7 (13.2%)	3 (37.5%)	
Sometimes	21 (34.4%)	19 (35.8%)	2 (25.0%)	
**Condom use with sex workers sexual partner in the last 12 months**	22 (100%)	17 (100%)	5 (100%)	
Always	11 (50.0%)	9 (52.9%)	2 (40.0%)	*0.367*
Never	8 (36.4%)	5 (29.4%)	3 (60.0%)	
Sometimes	3 (13.6%)	3 (17.6%)	---	
**Did you make tattoos?**	138 (100%)	92 (100%)	46 (100%)	
Yes	49 (35.5%)	35 (38.0%)	14 (30.4%)	*0.452*
No	89 (64.5%)	57 (62.0%)	32 (69.6%)	
**Did you share needles for injecting drugs?**	131 (100%)	86 (100%)	45 (100%)	
Yes	4 (3.1%)	4 (4.7%)	---	*0.298*
No	127 (96.9%)	82 (95.3%)	45 (64.3%)	
**Were invasive medical procedures performed?**	144 (100%)	93 (100%)	51 (100%)	
Yes	66 (45.8%)	43 (46.2%)	23 (45.1%)	*0.896*
No	78 (54.2%)	50 (53.8%)	28 (54.9%)	
**Have you had blood transfusions?**	133 (100%)	87 (100%)	46 (100%)	
Yes	20 (15.0%)	9 (10.3%)	11 (23.9%)	***0.044***
No	113 (85.0%)	78 (89.7%)	35 (76.1%)	

^a^ *p*-value: chi-square test or Fisher’s exact test.

**Table 3:** Diagnosis of HIV and sexually transmitted infections related to BESTHOPE study participants collected between September 2014 and December 2019.

	Total	Men	Women	*p* Value ^a^
	N (%)	N (%)	N (%)	
**STI diagnosis**	139 (100%)	89 (100%)	50 (100%)	
Yes	43 (30.9%)	36 (40.4%)	7 (14.0%)	***0.001***
No	96 (69.1%)	53 (59.6%)	43 (86.0%)	
**Date of last STI diagnosis**	76 (100%)	55 (100%)	21 (100%)	
<1 year	39 (51.3%)	29 (52.7%)	10 (47.6%)	***0.049***
1–5 years	22 (28.9%)	19 (34.5%)	3 (14.3%)	
≥5 years	5 (6.6%)	3 (5.5%)	2 (9.5%)	
Doesn’t know	10 (13.2%)	4 (7.3%)	6 (28.6%)	
**Hepatitis B diagnosis**	30 (100%)	19 (100%)	11 (100%)	
No	17 (56.7%)	12 (63.2%)	5 (45.5%)	*0.454*
Yes	13 (43.3%)	7 (36.8%)	6 (54.5%)	
**Hepatitis C diagnosis**	23 (100%)	16 (100%)	7 (100%)	
No	20 (87.0%)	13 (81.3%)	7 (100%)	*0.526*
Yes	3 (13.0%)	3 (18.8%)	-	
**Date of last HIV negative diagnosis**	135 (100%)	92 (100%)	43 (100%)	
<1 year	68 (50.4%)	46 (50.0%)	22 (51.2%)	*0.606*
1–5 years	47 (34.8%)	34 (37.0%)	13 (30.2%)	
≥5 years	20 (14.8%)	12 (13.0%)	8 (18.6%)	
**Date of HIV infection**	87 (100%)	69 (100%)	18 (100%)	
<1 year	39 (44.8%)	32 (46.4%)	7 (38.9%)	***0.184***
1–5 years	42 (48.3%)	34 (49.3%)	8 (44.4%)	
≥5 years	6 (6.9%)	3 (4.3%)	3 (16.7%)	
**Transmission route self-reported**	158 (100%)	101 (100%)	57 (100%)	
Sexual transmission route	122 (77.2%)	83 (82.2%)	39 (68.4%)	***0.124***
Others transmission route	5 (3.2%)	3 (3.0%)	2 (3.5%)	
Doesn’t know	31 (19.6%)	15 (14.9%)	16 (28.1%)	
**HIV serology of sexual partners**	135 (100%)	91 (100%)	44 (100%)	
HIV positive	40 (29.6%)	29 (31.9%)	11 (25.0%)	*0.628*
HIV negative	20 (14.8%)	14 (15.4%)	6 (13.6%)	
Doesn’t know	75 (55.6%)	48 (52.7%)	27 (61.4%)	
**If sexual transmission, which was the type of sexual partner**	100 (100%)	66 (100%)	34 (100%)	
Occasional	48 (48.0%)	42 (63.6%)	6 (17.5%)	***<0.001***
Regular	50 (50.0%)	22 (33.3%)	28 (82.4%)	
Sex worker	2 (2.0%)	2 (3.0%)	---	
**Were you having antiretroviral therapy?**	161 (100%)	101 (100%)	60 (100%)	
Yes	120 (74.5%)	79 (78.2%)	41 (68.3%)	*0.338*
No	38 (23.6%)	20 (19.8%)	18 (30.0%)	
Doesn’t know	3 (1.9%)	2 (2.0%)	1 (1.7%)	
**Was your sexual partner having antiretroviral therapy?**	158 (100%)	102 (100%)	56 (100%)	
Yes	12 (7.6%)	11 (10.8%)	1 (1.8%)	***0.115***
No	32 (20.3%)	19 (18.6%)	13 (23.2%)	
Doesn’t know	114 (72.2%)	72 (70.6%)	42 (75.0%)	
**CD4+ cell count (cell/mm^3^)**	258 (100%)	173 (100%)	85 (100%)	
CD4+ ≤ 350 cells/µL	142 (55.0%)	92 (53.2%)	50 (58.8%)	*0.426*
CD4+ > 350 cells/µL	116 (45.0%)	81 (46.8%)	35 (41.2%)	
CD4 baseline (Median; IQR)	329.00 (158.75–505.0)	343.0 (164.0–512.5)	307.0 (150.0–489.5)	*0.607 ^b^*

^a^ *p*-value: chi-square test or Fisher’s exact test; ^b^ *p*-value: *T*-test or nonparametric Man-Whitney Wilcoxon test.

**Table 4:** Determinants associated with an STI in the last 12 months (N = 141; OR: odds ratios; aOR: adjusted odds ratio; CI: confidence interval).

	OR (95% IC)	*p*-Value	aOR (95% IC)	*p*-Value
**Sex**				
Male	Ref		Ref	
Female	0.240 (0.097–0.592)	**0.002**	1.363 (0.325–5.710)	0.672
**Age groups**				
18–24 years	Ref			
25–44 years	1.181 (0.374–3.729)	0.776		
45–63 years	0.629 (0.168–2.346)	0.490		
>63 years	1.100 (0.080–15.153)	0.943		
**Country of Origin**				
Sub-Saharan Africa	Ref		Ref	
Brazil and other Latin American countries	5.941 (2.507–14.079)	**<0.001**	1.880 (0.526–6.722)	0.332
Western and Eastern Europe	4.917 (0.941–25.681)	0.059	2.518 (0.353–17.986)	0.357
Other countries	13.111 (1.075–159.858)	**0.044**	7.060 (0.365–136.614)	0.196
**Civil Status**				
Single	Ref		Ref	
Married	0.304 (0.125–0.739)	**0.009**	0.559 (0.186–1.679)	0.300
Divorced	0.422 (0.107–1.656)	0.216	0.646 (0.116–3.600)	0.618
Widowed	---	---	---	---
**Route of transmission**				
Heterosexual	Ref		Ref	
MSM	9.221 (3.960–21.469)	**<0.001**	5.695 (1.249–25.978)	**0.025**
IDU	6.455 (0.376–110.877)	**0.199**	2.489 (0.098–63.462)	0.581
Other/Unknown	---	---	---	---
**School Level**				
Third level (9th degree)	Ref		Ref	
Secondary (12nd degree)	4.392 (1.418–13.603)	**0.010**	1.353 (0.322–5.683)	0.679
Advanced Technical	3.100 (0.673–14.278)	**0.147**	1.202 (0.196–7.367)	0.842
Higher education	4.392 (1.418–13.603)	**0.010**	0.958 (0.221–4.155)	0.954
**Current Occupation**				
Employed	Ref			
Unemployed	0.646 (0.273–1.533)	0.322		
**Current Income**				
Very insufficient	0.923 (0.273–3.123)	0.897		
Insufficient	Ref			
Sufficient	1.353 (0.583–3.143)	0.482		
More than sufficient	1.292 (0.209–8.000)	0.783		

## Data Availability

The data presented in this study are available on request from the corresponding author.

## Supplementary Materials

The following supporting information can be downloaded at: https://www.mdpi.com/article/10.3390/pathogens13070598/s1, Supplementary Table S1: Ethical approvals from the institutions participating in the BESTHOPE study with dates of approval.

## Appendix A

BESTHOPE Study Group: Margarida Serrado (Unidade Imunodeficiência, Hospital Pulido Valente—Unidade Local de Saúde Santa Maria); Raquel Pinho and Liliana Pedro (Serviço de Medicina 3, Hospital de Portimão, Centro Hospitalar do Algarve, Portimão, Portugal); Maria José Manata (Serviço de Doenças Infeciosas, Hospital Curry Cabral, Centro Hospitalar Universitário Lisboa Central—Unidade Local de Saúde São José); Catarina Rodrigues and Joana Simões (Serviço de Medicina 1.4, Hospital de São José, Centro Hospitalar Universitário de Lisboa Central, Lisboa, Portugal); José Saraiva da Cunha (Serviço de Prevenção e Controlo de Infeções e de Resistências aos Antimicrobianos da Unidade Local de Saúde de Coimbra, Coimbra, Portugal); Bianca Ascenção (Serviço de Infeciologia, Centro Hospitalar de Setúbal, Setúbal, Portugal); Teresa Baptista (Serviço de Doenças Infeciosas, Hospital de Egas Moniz, Centro Hospitalar de Lisboa Ocidental, Lisboa, Portugal); Daniel Simões (Grupo de Ativistas em Tratamentos (GAT), Lisboa, Portugal); Rui Sarmento e Castro (Serviço de Infeciologia, Centro Hospitalar do Porto, Porto, Portugal); Sofia Nunes (Serviço de Infeciologia, Hospital de Aveiro, Centro Hospitalar Baixo Vouga, Aveiro, Portugal); Maria João Aleixo (Serviço de Infeciologia, Hospital Garcia da Orta, Almada, Portugal); Micaela Caixeiro (Serviço de Infeciologia, Hospital Dr. Fernando da Fonseca, Amadora, Portugal); Ana Rita Silva and Paulo Rodrigues (Serviço de Infeciologia, Hospital Beatriz Ângelo, Loures, Portugal); Carmela Piñero, Cátia Caldas and Jorge Soares (Serviço de Doenças Infeciosas, Unidade Local de Saúde de São João, Porto, Portugal); Elisabete Gomes da Silva (Unidade Local de Saúde do Baixo Alentejo, Hospital José Joaquim Fernandes, Beja, Portugal); Isabel Diogo (Laboratório de Biologia Molecular (LMCBM, SPC, ULSLO-HEM), Lisboa, Portugal); Carmo Koch and Fátima Monteiro (Centro de Biologia Molecular, Serviço de Imunohemoterapia, Unidade Local de Saúde de São João, Porto, Portugal); Célia Morais and Fernando Rodrigues (Serviço de Patologia Clínica, Centro Hospitalar e Universitário de Coimbra, Coimbra, Portugal); Guilhermina Gaião (Serviço de Patologia Clínica, Hospital de Sta Maria, Centro Hospitalar Universitário de Lisboa Norte, Portugal); Helena Ramos (Serviço de Patologia Clínica, Centro Hospitalar do Porto, Porto, Portugal); Olga Costa and Rita Côrte-Real^29^ (Serviço de Patologia Clínica, Biologia Molecular, Centro Hospitalar Universitário de Lisboa Central, Lisboa, Portugal).

## References

[1] Global HIV & AIDS Statistics—Fact Sheet|UNAIDS [Internet]. https://www.unaids.org/en/resources/fact-sheet.

[2] Sexually Transmitted Infections (STIs) [Internet]. https://www.who.int/news-room/fact-sheets/detail/sexually-transmitted-infections-(stis).

[3] Baggaley R.F., Zenner D., Bird P., Hargreaves S., Griffiths C., Noori T., Friedland J.S., Nellums L.B., Pareek M. (2022). Prevention and treatment of infectious diseases in migrants in Europe in the era of universal health coverage. Lancet Public Health.

[4] Ross J., Cunningham C.O., Hanna D.B. (2018). HIV outcomes among migrants from low-income and middle-income countries living in high-income countries. Curr. Opin. Infect. Dis..

[5] Zheng Y., Yu Q., Lin Y., Zhou Y., Lan L., Yang S., Wu J. (2022). Global burden and trends of sexually transmitted infections from 1990 to 2019: An observational trend study. Lancet Infect. Dis..

[6] Wi T.E., Ndowa F.J., Ferreyra C., Kelly-Cirino C., Taylor M.M., Toskin I., Kiarie J., Santesso N., Unemo M. (2019). Diagnosing sexually transmitted infections in resource-constrained settings: Challenges and ways forward. J. Int. AIDS Soc..

[7] Prevention—STD Information from CDC [Internet]. https://www.cdc.gov/std/prevention/default.htm.

[8] Cohen M.S., Council O.D., Chen J.S. (2019). Sexually transmitted infections and HIV in the era of antiretroviral treatment and prevention: The biologic basis for epidemiologic synergy. J. Int. AIDS Soc..

[9] (2021). Relatório de Imigração, Fronteiras e Asilo. Serviço dos Estrangeiros e Fronteira [Internet]. https://sefstat.sef.pt/Docs/Rifa2021.pdf.

[10] (2022). Portugal. Ministério da Saúde. Direção-Geral da Saúde/Instituto Nacional de Saúde Doutor Ricardo Jorge. Infeção por VIH em Portugal—2022. Lisboa: DGS/INSA. https://repositorio.insa.pt/bitstream/10400.18/8383/3/Relato%cc%81rio_VIH_Portugal_2022_Vjulho2023.pdf.

[11] Jardim Santos C., Gomes B., Ribeiro A.I. (2020). Mapping Geographical Patterns and High Rate Areas for Sexually Transmitted Infections in Portugal: A Retrospective Study Based on the National Epidemiological Surveillance System. Sex Transm. Dis..

[12] Fonseca S., Lacerda L., Teixeira C., Reis-Melo A., Tavares M. (2022). Sexually transmitted infections in Portuguese adolescents. Pediatría (Engl. Ed.).

[13] Relatório de imigração Fronteiras e Asilo 2022. Serviço dos Estrangeiros e Fronteira [Internet]. https://www.sef.pt/pt/Documents/RIFA2022%20vF2a.pdf.

[14] Dias S., Gama A., Abrantes P., Gomes I., Fonseca M., Reigado V., Simões D., Carreiras E., Mora C., Pinto Ferreira A. (2020). Patterns of Sexual Risk Behavior, HIV Infection, and Use of Health Services Among Sub-Saharan African Migrants in Portugal. J. Sex Res..

[15] Dias S., Gama A., Severo M., Barros H. (2011). Factors associated with HIV testing among immigrants in Portugal. Int. J. Public Health.

[16] Dias S., Marques A., Gama A., Martins M. (2014). HIV Risky Sexual Behaviors and HIV Infection Among Immigrants: A Cross-Sectional Study in Lisbon, Portugal. Int. J. Environ. Res. Public Health.

[17] Koschollek C., Kuehne A., Müllerschön J., Amoah S., Batemona-Abeke H., Dela Bursi T., Mayamba P., Thorlie A., Mputu Tshibadi C., Wangare Greiner V. (2020). Knowledge, information needs and behavior regarding HIV and sexually transmitted infections among migrants from sub-Saharan Africa living in Germany: Results of a participatory health research survey. PLoS ONE.

[18] Maharajh R., Haffejee F. (2021). Exploring male condom use among women in South Africa: A review of the literature. Afr. J. AIDS Res..

[19] Ntshiqa T., Musekiwa A., Mlotshwa M., Mangold K., Reddy C., Williams S. (2018). Predictors of male condom use among sexually active heterosexual young women in South Africa, 2012. BMC Public Health.

[20] van Loggerenberg F., Dieter A.A., Sobieszczyk M.E., Werner L., Grobler A., Mlisana K. (2012). HIV Prevention in High-Risk Women in South Africa: Condom Use and the Need for Change. Getz WM, editor. PLoS ONE.

[21] Dias S., Gama A., Tavares A.M., Reigado V., Simões D., Carreiras E., Mora C., Pinto Ferreira A. (2019). Are Opportunities Being Missed? Burden of HIV, STI and TB, and Unawareness of HIV among African Migrants. Int. J. Environ. Res. Public Health.

[22] Women and Girls Still Vulnerable to HIV Due to Gender Inequality: UNAIDS|UN News [Internet]. https://news.un.org/en/story/2020/03/1058751.

[23] Sentís A., Martin-Sanchez M., Arando M., Vall M., Barbera M.J., Ocaña I., Cordón A.G., Alsina M., Martin-Ezquerra G., Knobel H. (2019). Sexually transmitted infections in young people and factors associated with HIV coinfection: An observational study in a large city. BMJ Open.

[24] Pérez-Morente M.Á., Cano-Romero E., Sánchez-Ocón M.T., Castro-López E., Jiménez-Bautista F., Hueso-Montoro C. (2017). Sexuality Risk Factors among People with Suspect of Sexually Transmitted Disease. Rev. Esp. Salud. Publica.

[25] Fasciana T., Capra G., Di Carlo P., Calà C., Vella M., Pistone G., Colomba C., Giammanco A. (2021). Socio-Demographic Characteristics and Sexual Behavioral Factors of Patients with Sexually Transmitted Infections Attending a Hospital in Southern Italy. Int. J. Environ. Res. Public Health.

[26] Aral S.O. (2001). Sexually transmitted diseases: Magnitude, determinants and consequences. Int. J. STD AIDS.

