# Supplementary material for: Sociodemographic, Clinical, and Behavioral Factors Associated with Sexual Transmitted Infection among HIV-1 Positive Migrants in Portugal: Are There Differences between Sexes?
Source: Pathogens. 2024 Jul 19;13(7):598. doi: 10.3390/pathogens13070598 (PMC11280352; doi:10.3390/pathogens13070598)
Supplement: Supplementary file 1 [file pathogens-13-00598-s001.zip › pathogens-3065944-supplementary.pdf]

## Ethical committees approval- Supplementary Materials

**Supplementary Table S1-** Ethical approvals from the institutions participating in the BESTHOPE study with dates of approval.

| Site | Number of document | Date of approval |
|---|---|---|
| Centro Hospitalar do Algarve | 2062/2015 | 12-10-2015 |
| Centro Hospitalar do Baixo Vouga - Aveiro | 164/AD | 25-05-2015 |
| Centro Hospitalar de Lisboa Central | 233/2015 | 25-09-2015 |
| Centro Académico de Medicina de Lisboa | 187/16 | 07-11-2016 |
| Centro Hospitalar Lisboa-Ocidental | 40/CES-2017 | 06-03-2017 |
| Centro Hospitalar do Porto | 2015.165 (144-DEFI/134-CES) | 02-09-2015 |
| Centro Hospitalar de Setúbal | 11/16 | 10-03-2016 |
| Centro Hospitalar de São João | 241/15 | 09-12-2015 |
| Hospital Beatriz Ângelo | 1244/2015_MJHEB | 08-09-2015 |
| Hospital de Santarém | | 14-12-2015 |
| Hospital Prof. Dr. Fernando Fonseca | | 29-04-2016 |
| Unidade Local de Saúde do Baixo Alentejo | 02/2016 | 17-06-2016 |
| Unidade Local de Saúde de Matosinhos- Hospital Pedro Hispano | 080/CE/JAS | 11-12-2015 |
